# The effect of recurrent application of concentrated platelet-rich fibrin inside the extraction socket on the hard and soft tissues. a randomized controlled trial

**DOI:** 10.1186/s12903-023-03400-5

**Published:** 2023-09-19

**Authors:** Cezar Lahham, Mahmoud Abu Ta’a, Elias Lahham, Saleem Michael, Wael Zarif

**Affiliations:** 1 Department of Dental Science, Faculty of graduate studies, Arab American University, Ramallah, Palestine; 2 Department of Medicine, Al-Quds University, Abu Dis, Palestine; 3 Department of Nursing and Health Science, Bethlehem University, Bethlehem, Palestine; 4 Department of Oral and Maxillofacial Surgery, Hama National Hospital, Hama, Syria

**Keywords:** Recurrent injections, Platelet-rich fibrin, Ridge alterations, Tooth extraction, Ridge preservation

## Abstract

**Background:**

Platelet-rich fibrin (PRF) is commonly used for ridge preservation following tooth extraction. However, its effectiveness diminishes over a period of two weeks as it is resorbed and loses its biological activities. Therefore, this clinical study aims to evaluate the effect of recurrent application of concentrated PRF (C-PRF) inside the extraction socket on the hard and soft tissue alterations.

**Methods:**

Twenty patients requiring single tooth extraction and replacement with a dental implant were randomized into one of two ridge preservation approaches: Advanced PRF plus alone (Control group) or advanced PRF plus with the recurrent application of a C-PRF inside the socket every two weeks for 2 months (four times). The ridge width, the ridge height, and the soft tissue thickness were assessed clinically at the baseline and reassessed after 3 months from tooth extraction during implant surgery. Then the amount of hard tissue loss and soft tissue alterations were calculated.

**Results:**

There was a statistically significant difference in the amount of hard tissue loss between groups in the third month. The amount of horizontal ridge loss for the control and test groups were 2.9 ± 0.7 mm and 1.9 ± 0.5 mm, respectively (p-value < 0.05). The vertical bone loss for control and test groups were 1.8 ± 0.5 mm and 1.0 ± 0.3 mm, respectively (p-value < 0.05). Additionally, for the soft tissue thickness, there was no statistical difference between the groups (p-value > 0.05).

**Conclusion:**

Within the limitations of this study, the recurrent application of C-PRF in the extraction socket could decrease the amount of ridge alteration following tooth extraction and may play a role in the bone regeneration procedures.

**Trial registration:**

Registered on ClinicalTrials.gov (ID: NCT05492357, on 08/08/2022).

## Background

Tooth extraction is one of the most common procedures performed in dental clinics. Although immediate implant placement has become popular following tooth extraction, it is not indicated in all cases and does not prevent physiological bone resorption and soft tissue alterations [1, 2]. It has been observed that the ridge width reduction could reach 50% at the extraction site within 12 months post-extraction [3]. Additionally, about 67% of the overall changes happened during the first 12 weeks following the extraction [3]. These results could be lower than the actual percentage of bone loss by 2-3.5 times [4]. In a systematic review, it was observed that the horizontal and vertical bone loss following tooth extraction are 3.55 ± 0.9 mm and 2.15 ± 1.75 mm, respectively [5]. Although the biological process of bone resorption post-extraction cannot be arrested, alveolar ridge preservation (ARP) modalities play a significant role in reducing the rate of physiologic bone resorption that occurs as a consequence of tooth extraction to facilitate dental replacement therapy [6].

Platelet-rich fibrin (PRF) is apart from natural blood product that’s produced after blood centrifugation. It contains concentrated platelets, growth factors, and cytokines [7]. Therefore, it represents one of the most common biomaterials used for socket augmentation, because it is self-sourced with a relatively low cost, provides a high rate of bone regeneration, which reaches 88% within 8 weeks, and decreases post-operative pain [8–10]. Additionally, it does not have absolute contraindications. PRF can be prepared in two forms, solid and liquid. The difference in PRF consistency is related to the selected preparation protocol and the tube that has been used [11]. Advanced platelet-rich fibrin plus (A-PRF+) is the last generation of solid-form PRF, with its approved clinical safety and efficacy in preclinical and clinical trials when compared to other PRF generations, A-PRF + interestingly showed a significant increase in growth factor release of TGF-b1, PDGF-AA, PDGF-AB, PDGF-BB, VEGF, IGF, and EGF. which have been shown to promote bone tissue regeneration and wound healing [12–14].

Concentrated platelet-rich fibrin (C-PRF) is the last generation of liquid-form PRF. It is mainly used in dermatology and bone augmentation procedures. While the use of C-PRF in bone regeneration is usually done once during the surgical phase, it is recommended to be used many times for skin and hair regeneration [15].

Although A-PRF + has many clinical benefits, its effectiveness diminishes over a period of two weeks as it is resorbed and loses its biological activities [12, 16, 17]. Therefore, this clinical study aims to evaluate the effect of recurrent application of C-PRF injections inside the extraction socket on hard tissue dimensions, in addition to soft tissue thickness at the extraction site.

## Materials and methods

### Study design, patient recruitment, and randomization

This clinical study was conducted between June 15th 2022 and October 18th 2022, in accordance with the Helsinki Declaration of 1975, as revised in 2000. Ethical approval was obtained from the institutional Ethics Committee of Arab American University and the Palestinian Health Research Council [PHRC/HC/1151/22] on May 26th 2020. The ClinicalTrials.gov registration number for this study is NCT05492357 on 08/08/2022.

**Fig. 1:**
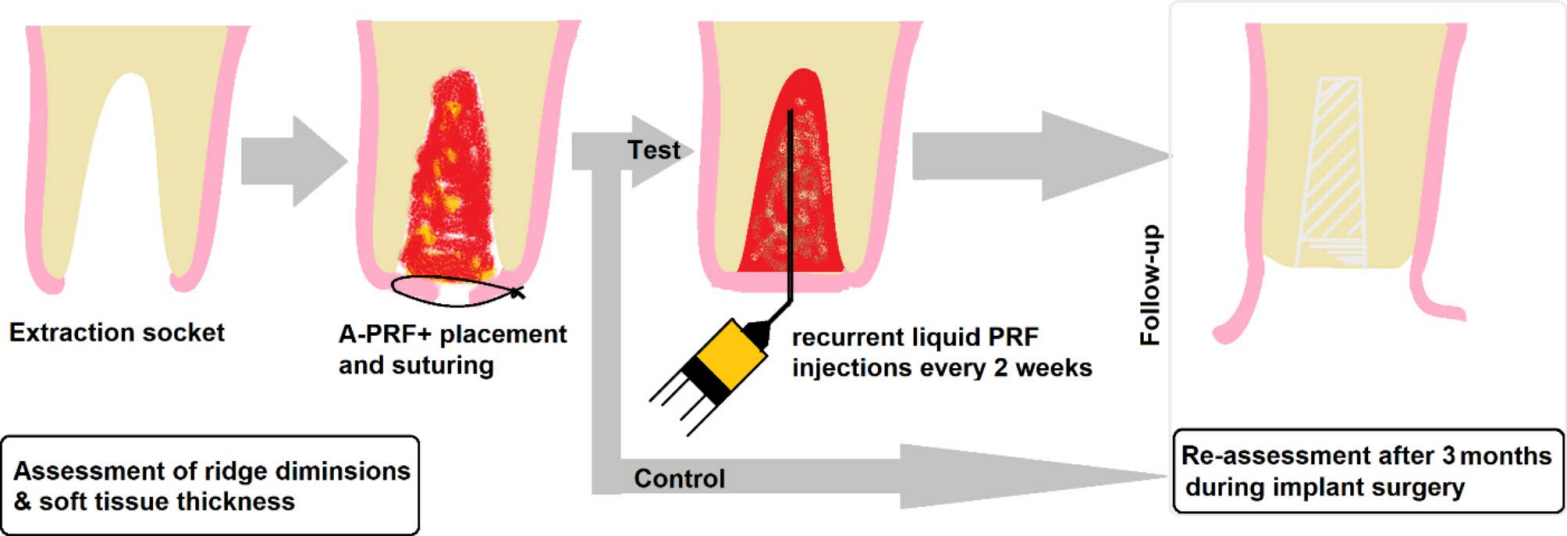
Study plan

Patient’s recruitment was done in the Department of Periodontology and Implant Dentistry at Arab American University of Palestine. All patients signed a written informed consent before starting the procedure. Additionally, consent to participate in this clinical trial was also obtained from all patients. The inclusion criteria for the study were: A single maxillary and mandibular non-molar tooth requiring extraction, alveolar bone level (pre-assessed using periapical radiographs and ensured clinically following tooth extraction and small flap elevation) more than 67% of the root length, and age ranges from 20 to 65 years. The exclusion criteria included: bony fenestration of the socket wall or absence of the buccal plate (assessed clinically following tooth extraction using probe and direct vision), acute infection, periodontally compromised tooth, and any systemic disease which may affect the healing process [Table 1]. All patients were randomly allocated to either advanced platelet-rich fibrin plus (A-PRF+) alone or A-PRF + with a recurrent application of C-PRF injections (Fig. 1). The CONSORT guidelines for reporting randomized controlled trials were followed in this study.

**Table 1:** Inclusion and exclusion criteria

Inclusion criteria	Exclusion criteria
A single maxillary and mandibular non-molar tooth requiring extraction	Presence of bony fenestration of the socket wall or absence of the buccal plate
Alveolar bone level more than 67% of the root length	Presence of acute infection and periodontally compromised teeth
Age ranges from 20 to 65 years	Presence of any systemic disease

At first, 20 patients were divided according to their gender. Then men and women were equally and randomly separated into the previously mentioned groups (stratified randomization [18]). Randomization was performed by using a random team generator software application (Keamk®) as it avoids selection bias.

### Sample size estimation

The sample size for this study was estimated using t-tests. The power sample size was calculated to detect a difference of 0.5 mm in vertical and horizontal bone resorption between the test and control groups. A power analysis in G-Power suggested a sample size of 16 participants, assuming 0.90 power with an α = 0.05. However, the sample size was increased to 20 patients due to the risk of drop-out during follow-up.

The sample size calculation was done using G-Power software version 3.1.9.7 [19].

### Pre-extraction preparation

Before extraction, periapical radiographs were taken to assess the level of the alveolar bone. Informed consent was taken from all participants. 20 ml of venous blood was collected from each patient using two sterile vacutainer red-cap tubes (10 ml each). The venous blood was spun in the centrifugation machine (PRF DUO® Centrifuge, France) at 1300 rpm for 8 min (relative centrifugal force: 208 g) in accordance to Fujioka-Kobayashi et al. protocol. [12] After the completion of centrifugation, caps were removed for 5 min to induce more clotting Then, the fibrin clots were extracted from the tubes and lightly compressed to form a PRF plug.

### Extraction procedures and clinical parameters assessment

After the administration of lidocaine HCl 2% and epinephrine 1:100,000, soft tissue thickness (ST) was assessed in the mid-buccal region of the tooth to be extracted at the level of alveolar crest (2.5 mm below the imaginary line connecting zenith points of the adjacent teeth) using a periodontal probe (UNC-15). Following that, a small flap was done, tooth extraction was performed with an atraumatic procedure, and the socket was carefully curetted and irrigated using normal saline.

**Fig. 2:**
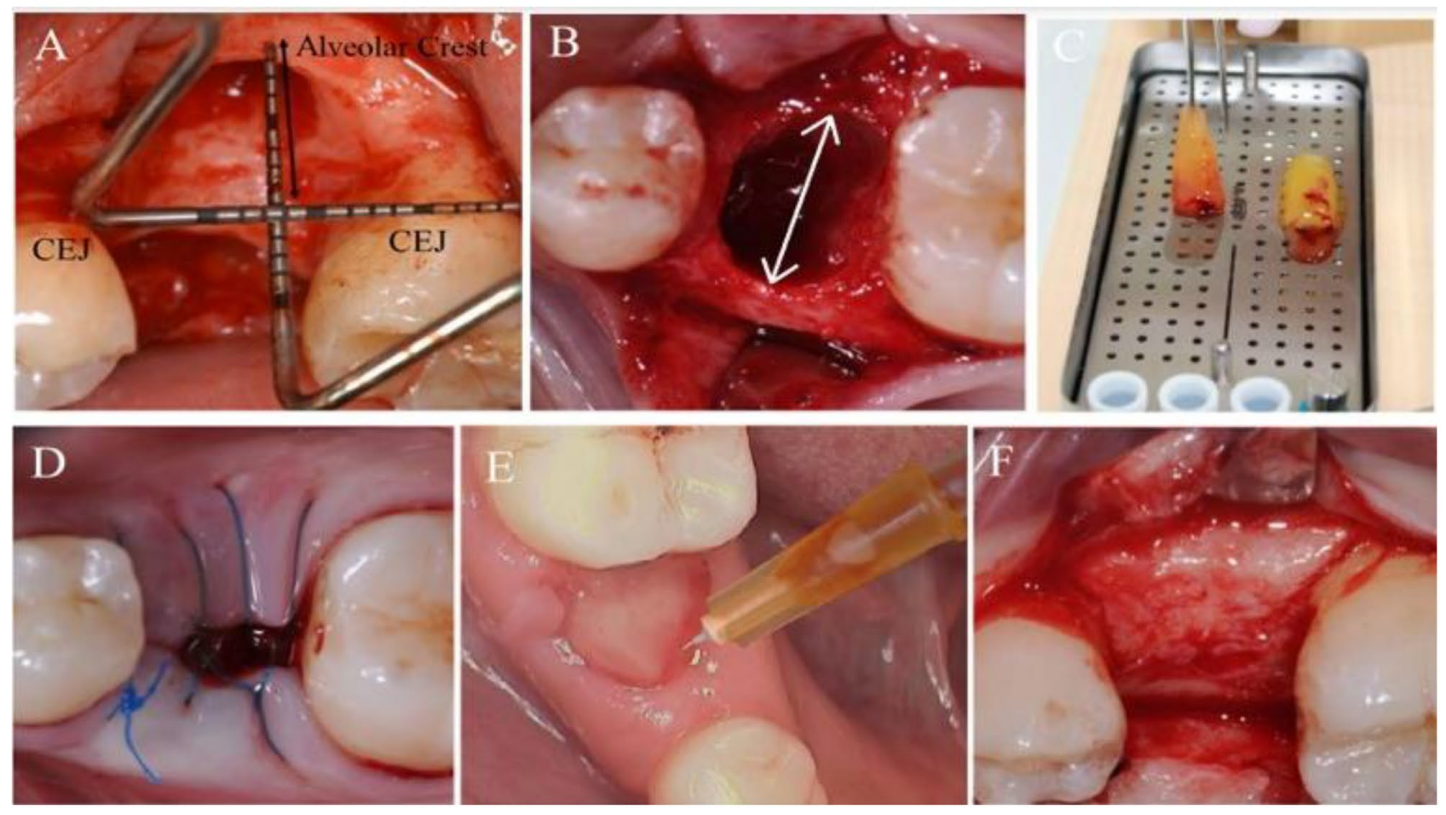
**(A)** Ridge height assessment at the baseline in reference to cemento-enamel junction (CEJ); **(B)** Ridge width in the crestal part; **(C)** A-PRF+; **(D)** Suturing over A-PRF+; **(E)** C-PRF injection using a 25-gauge needle; **(F)** Ridge dimensions after 3 months

The level of the alveolar crest was measured in reference to the cement-enamel junction (CEJ) of the adjacent teeth using a periodontal probe (UNC-15). Whereas the horizontal ridge dimension was assessed clinically using the same periodontal probe (Fig. 2).

### A-PRF + placement inside the extraction socket

In all patients, the socket was filled with A-PRF + membranes carved to fit the extraction site and placed inside the socket. After that, A-PRF + was secured in place using a 4.0 nylon suture. All the surgeries were performed by the same periodontist (C.L.).

### Post-operative instructions

All the participants were instructed to avoid brushing at the surgical site for 2 weeks. Moreover, they rinsed twice daily with chlorhexidine mouth rinse at 0.2% (Gargarol ®) after surgery for 2 weeks. Furthermore, each patient was prescribed 400 mg ibuprofen to be taken if needed. Finally, sutures were removed after 2 weeks from the extraction.

### Recurrent application of C-PRF for the test group

Two weeks following a tooth extraction, the test group started receiving C-PRF injections inside the extraction socket. Immediately following sutures removal, 18 ml venous blood was collected in two sterile vacutainer white tubes (VACUETTE®, 9 ml each). The venous blood was spun in the centrifugation machine (PRF DUO® Centrifuge, France) at 2500 rpm for 8 min (relative centrifugal force: 700 g) [20]. After completion of centrifugation, the concentrated PRF layer which is 0.3–0.5 ml (immediately above the red layer in the tube) was collected using a needle 18 G x 1.5 in. Then 0.5-1 ml of C-PRF was applied deep inside the socket using a needle 25 G X 5/8 in. This procedure was repeated every two weeks four times.

### Re-entry after 3 months for reassessment and implant placement

After the administration of lidocaine HCl 2% and epinephrine 1:100,000, ST was reassessed from the mid-buccal region of the extraction site at the level of alveolar crest (2.5 mm below the imaginary line connecting zenith points of the adjacent teeth) using a periodontal probe (UNC-15). Then, a mid-crestal incision and flap elevation was performed. The amount of vertical bone loss was calculated after measuring the distance between the alveolar crest and the CEJ. The amount of horizontal bone loss was calculated after measuring the ridge width in the third month. Then, implant placement was done.

### Statistical analysis

Descriptive statistics for data analysis were expressed using a statistical tool package (IBM SPSS software version 23, Chicago, USA). Mean with standard deviations (Mean ± SD) were addressed to exhibit average bone loss. Paired t-tests for matched samples were employed to calculate the vertical and horizontal bone changes identified within each group (intragroup) from baseline to 3 months. The difference between groups was determined using an independent samples t-test. Non-parametric tests were applied to assess Soft tissue dimensional alterations, due to unmet criteria of normal distribution. The P values were calculated using median values with an interquartile range. The Wilcoxon signed-rank test was used to compare the median values of clinical parameters at baseline and 3 months post-extraction within each group (intragroup). For intergroup changes from baseline and 3 months post-extraction, the Mann-Whitney U test was used. All statistical tests were carried out at a significant level of 5%.

## Results

A total of 20 patients (12 men, 8 women), who fulfilled the criteria were enrolled for the socket augmentation, and all of them completed the study. The extracted teeth were twenty (13 maxillary teeth and 7 mandibular teeth). No complications happened among the twenty cases. Of these, 10 patients (6 men and 4 women; mean age 48.70 ± 10.55 years; range, 27–63 years) were randomly assigned to A-PRF + alone (control group) and 10 patients (6 men and 4 women; mean age 49.10 ± 8.92 years; range, 26–58 years) were assigned to A-PRF + with the recurrent application of C-PRF (test group). [Table 2]

**Table 2:** Baseline demographic data

	Control group	Test Group
Mean age (years)	48.70 ± 10.55	49.10 ± 8.92
Gender	6 men and 4 women	6 men and 4 women
Extracted teeth	7 maxillary teeth and 3 mandibular teeth	6 maxillary teeth and 4 mandibular teeth

**Fig. 3:**
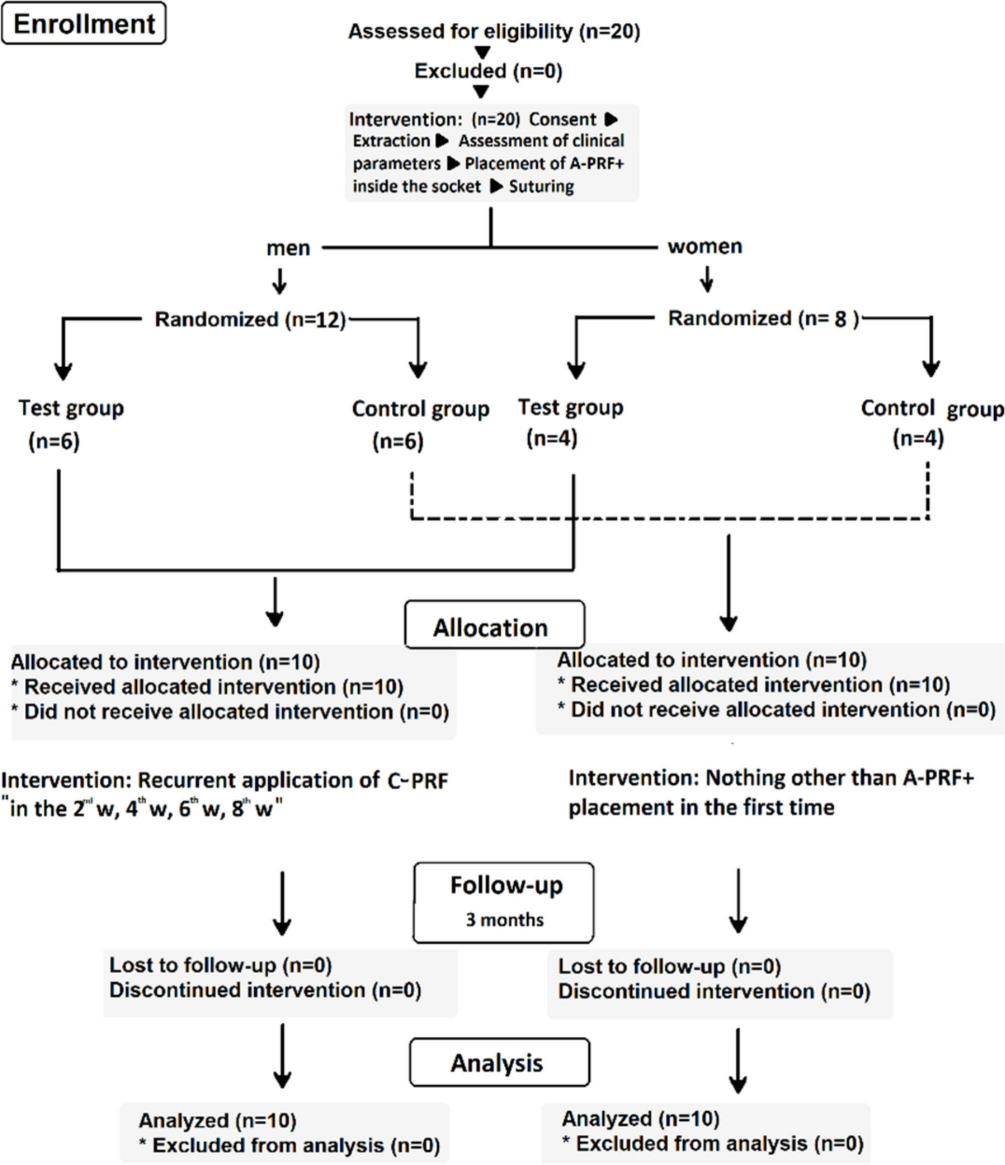
CONSORT flow diagram of this study

The clinical measurements of the extraction sockets, recorded at baseline and 3 months post-extraction were summarized in Table 3. (See the CONSORT flow diagram in Fig. 3)

**Table 3:** HRM: Horizontal ridge measurement, VRL: Vertical ridge level in reference to CEJ, ST: Soft tissue thickness

	Baseline (mean ± SD)	After 3 months (mean ± SD)	Difference (mean ± SD)	p Value (Within group)	p Value (Between groups)
HRM: Control	8.3 ± 1.2	5.4 ± 0.8	2.9 ± 0.7	0.16	* 0.01 (< 0.05)
HRM: Test	8.5 ± 1.4	6.6 ± 0.9	1.9 ± 0.5	0.29	
VRL: Control	2.5 ± 0.7	3.5 ± 0.3	1.8 ± 0.5	0.14	* 0.04 (< 0.05)
VRL: Test	2.8 ± 0.8	3.8 ± 0.5	1.0 ± 0.3	0.09	
ST: Control	1.5 ± 0.3	1.7 ± 0.5	0.2 ± 0.1	0.22	0.9 (> 0.05)
ST: Test	1.3 ± 0.2	1.5 ± 0.3	0.2 ± 0.1	0.17	

*: Statistically significant difference (p-Value < 0.05).

**Chart 1:**
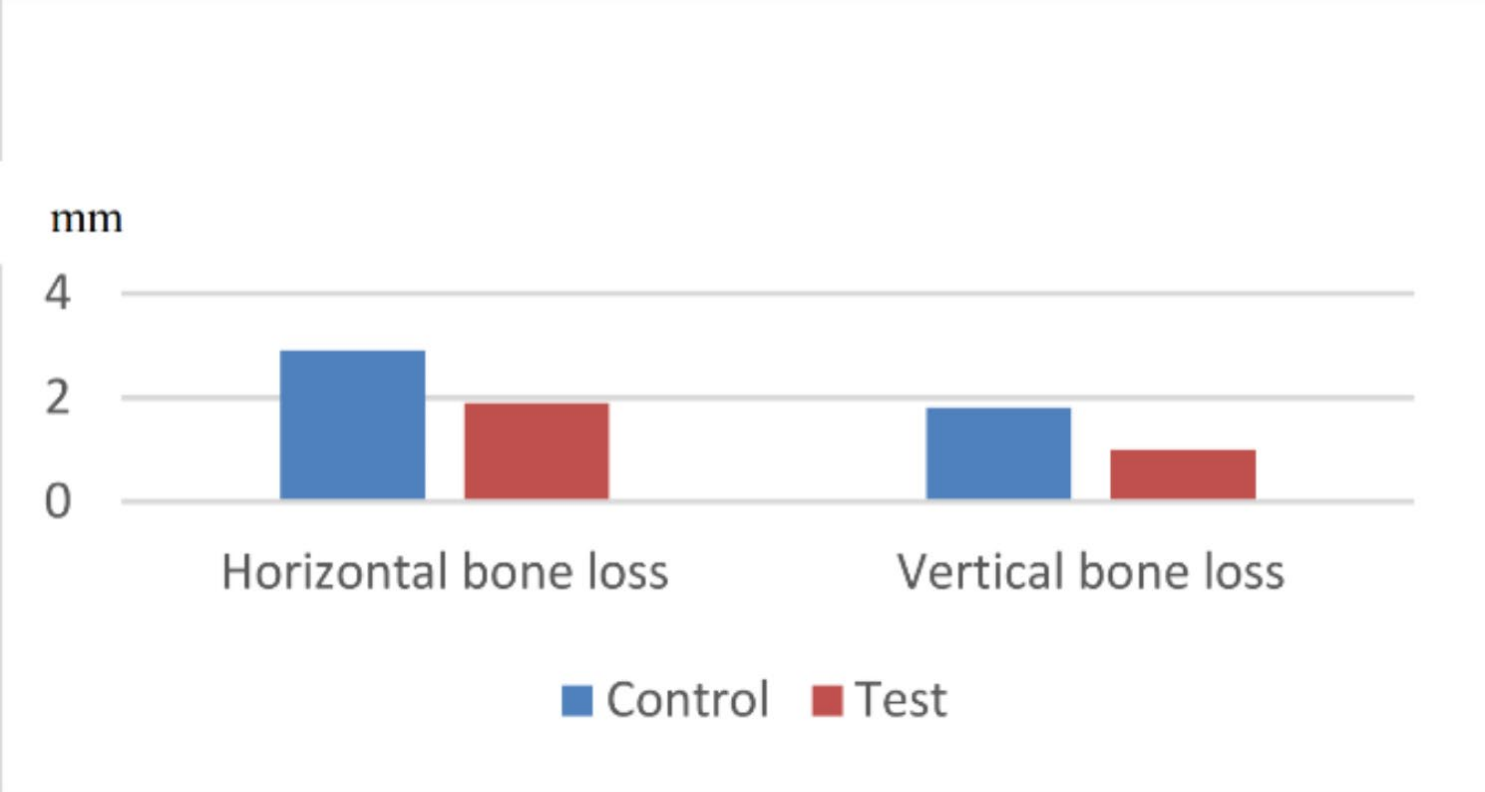
Amount of bone loss 3 months following tooth extraction

As reported in Table 2, there was a statistically significant difference in the amount of hard tissue loss between groups in the third month. The amount of horizontal ridge loss for the control and test groups were 2.9 ± 0.7 mm and 1.9 ± 0.5 mm, respectively (p-value < 0.05). The vertical bone loss for control and test groups were 1.8 ± 0.5 mm and 1.0 ± 0.3 mm, respectively (p-value < 0.05). Furthermore, there was no significant difference observed in soft tissue thickness between groups (p-value > 0.05) [Chart 1].

## Discussion

This randomized controlled trial was designed to evaluate the efficacy of recurrent application of C-PRF injections inside the extraction sockets on the hard and soft tissue dimensional alterations following tooth extraction. This study represents the first clinical study to evaluate the effectiveness of recurrent C-PRF injections for ridge preservation.

PRF is prepared from the patient’s blood without adding any materials. Therefore, it’s resorbed completely without leaving any negative effects. In contrast to PRF, socket augmentation with bone graft is more expensive, time consuming, and may leave some residual materials that can affect the osseointegration of the dental implant later on [21, 22]. Several studies have shown that PRF/bone graft mixture can reduce the dimensional changes of the alveolar ridge and decrease the amount of residual bone graft particles [23].

In this study, statistically significant differences in the horizontal and vertical dimensions were observed between the groups after 3 months of healing. Ridge preservation using A-PRF + with recurrent C-PRF injections demonstrated lower alveolar bone resorption than A-PRF + alone by 1.5 times. For the A-PRF + group in this study, the amount of horizontal and vertical bone loss was 2.9 mm and 1.8 mm, respectively. These results were comparable to Clark’s clinical trial results [24]. Many previous studies have shown the superiority of A-PRF + clinically compared to other PRF generations [25, 23, 26, 27]. The A-PRF + protocol uses lower g-forces with lower centrifugal duration, which has been shown to allow for greater release of growth factors and leukocytes from the clots in vitro [28, 29, 11].

Several studies have shown the positive effects of the recurrent application of liquid PRF injections for soft tissue healing and improvement [30, 31]. As it was observed, PRF diminished gradually over a period of 2 weeks, the recurrent application of liquid PRF can refill the place again with active cells that produce growth factors [31, 17]. In this study, recurrent C-PRF injections were performed every 2 weeks for a 2-month period. During this period, bone fill was gradually happening, and the needle became unable to enter more than half of the extraction socket in the second month.

There are many preparation protocols for liquid PRF. However, it was shown that C-PRF collected from the buffy coat layer following high centrifugation protocols represents a novel harvesting technique that was recently developed to extract higher concentrations of platelets/leukocytes. This protocol produces PRF with the greatest potential for cell migration and proliferation, with a higher level of TGF-β1, PDGF-AA, and EGF in comparison with other traditional preparation protocols [20]. Therefore, this new protocol was used in this study to reach the highest capacity of regenerative properties.

For soft tissue thickness in this study, there were no statistically significant differences between groups in the third month, and this result is comparable to Avila-Ortiz’s study results [32].

This clinical trial, however, had many limitations. Further studies with a larger sample size are recommended. Histological investigations are also recommended to assess bone quality following the recurrent application of C-PRF injections. Another limitation in this study was the use of a fixed-angle centrifuge instead of a horizontal centrifuge. Finally, we recommend using surgical templates and CBCT to ensure the accuracy of clinical outcomes.

## Conclusion

Within the limitations of this study, the recurrent application of C-PRF inside the extraction socket could decrease the amount of ridge alteration following tooth extraction. This finding could be of research interest for future studies related to ridge augmentation assisted by the recurrent application of C-PRF.

## Data Availability

The data used to support the findings of this study are included within the article.

## References

[1] Pietrokovski J, Massler M. Alveolar ridge resorption following tooth extraction. J Prosthet Dent. 1967;17(1). 10.1016/0022-3913(67)90046-7. 10.1016/0022-3913(67)90046-7 5224784

[2] Mahesh L, Calvo Guirado JL, Shukla S, Kumar VR, Kumar YR. Clinical and radiographic findings without the use of bone substitute materials in extraction sockets and delayed implant placement- A case series. J Oral Biology Craniofac Res. 2020;10(2). 10.1016/j.jobcr.2020.03.011. 10.1016/j.jobcr.2020.03.011 PMC7254475 32489812

[3] ‘Bone healing. And soft tissue contour changes following single-tooth extraction: a clinical and radiographic 12-month prospective study’. J Prosthet Dent. 2004;91(1). 10.1016/j.prosdent.2003.10.022. 12956475

[4] Araújo MG, Lindhe J. Dimensional ridge alterations following tooth extraction. An experimental study in the dog. J Clin Periodontol. 2005;32(2). 10.1111/j.1600-051X.2005.00642.x. 10.1111/j.1600-051X.2005.00642.x 15691354

[5] Ten Heggeler JMAG, Slot DE, Van Der Weijden GA. Effect of socket preservation therapies following tooth extraction in non-molar regions in humans: a systematic review. Clin Oral Implants Res. 2011;22(8). 10.1111/j.1600-0501.2010.02064.x. 10.1111/j.1600-0501.2010.02064.x 21091540

[6] Thalmair T, Fickl S, Schneider D, Hinze M, Wachtel H. Dimensional alterations of extraction sites after different alveolar ridge preservation techniques - a volumetric study. J Clin Periodontol. 2013;40(7). 10.1111/jcpe.12111. 10.1111/jcpe.12111 23647007

[7] Pavlovic V, Ciric M, Jovanovic V, Trandafilovic M, Stojanovic P. Platelet-rich fibrin: basics of biological actions and protocol modifications. Open Med (Poland). 2021;16(1). 10.1515/med-2021-0259. 10.1515/med-2021-0259 PMC7985567 33778163

[8] Pan J, et al. Effect of platelet-rich fibrin on alveolar ridge preservation: a systematic review. J Am Dent Assoc. 2019;150(9). 10.1016/j.adaj.2019.04.025. 10.1016/j.adaj.2019.04.025 31439204

[9] Yaprak E, Kasap M, Akpinar G, Islek EE, Sinanoglu A. Abundant proteins in platelet-rich fibrin and their potential contribution to wound healing: an explorative proteomics study and review of the literature. J Dent Sci. 2018;13(4). 10.1016/j.jds.2018.08.004. 10.1016/j.jds.2018.08.004 PMC6388803 30895150

[10] Alzahrani AA, Murriky A, Shafik S. Influence of platelet rich fibrin on post-extraction socket healing: a clinical and radiographic study. Saudi Dent J. 2017;29(4). 10.1016/j.sdentj.2017.07.003. 10.1016/j.sdentj.2017.07.003 PMC5634795 29033524

[11] Simões-Pedro M, Tróia PMBPS, Dos Santos NBM, Completo AMG, Castilho RM. and G. V. de O. Fernandes, ‘Tensile Strength Essay Comparing Three Different Platelet-Rich Fibrin Membranes (L-PRF, A-PRF, and A-PRF+): A Mechanical and Structural In Vitro Evaluation’, *Polymers*, vol. 14, no. 7, 2022, 10.3390/polym14071392. 10.3390/polym14071392 PMC9002533 35406263

[12] Fujioka-Kobayashi M, Miron RJ, Hernandez M, Kandalam U, Zhang Y, Choukroun J. Optimized platelet-rich fibrin with the low-speed Concept: growth factor release, Biocompatibility, and Cellular Response. J Periodontol. 2017;88(1). 10.1902/jop.2016.160443. 10.1902/jop.2016.160443 27587367

[13] CTRI/2017/12. /010879, ‘To assess the results of medicine made from patients extracted blood with/without medicine made from bone for preserving tooth socket after extraction- a randomized control trial’, https://trialsearch.who.int/Trial2.aspx?TrialID=CTRI/2017/12/010879, 2017.

[14] Machut K, Żółtowska A. Plasma Rich in Growth factors in the treatment of Endodontic Periapical Lesions in adult patients: 3-Dimensional analysis using Cone-Beam Computed Tomography on the outcomes of non-surgical endodontic treatment using A-PRF + and calcium hydroxide: a retrospective cohort study. J Clin Med. 2022;11(20). 10.3390/jcm11206092. 10.3390/jcm11206092 PMC9605098 36294413

[15] Dashore S, Chouhan K, Nanda S, Sharma A. Platelet-Rich Fibrin, Preparation and Use in Dermatology. Indian Dermatology Online Journal. 2021;12(7). 10.4103/idoj.idoj_282_21. 10.4103/idoj.idoj_282_21 PMC8664174 34976881

[16] Miron RJ, Pikos MA (2017). PRF as a barrier membrane in guided bone regeneration. Dent Today.

[17] Kobayashi E, et al. Comparative release of growth factors from PRP, PRF, and advanced-PRF. Clin Oral Invest. 2016;20(9). 10.1007/s00784-016-1719-1. 10.1007/s00784-016-1719-1 26809431

[18] Kernan WN, Viscoli CM, Makuch RW, Brass LM, Horwitz RI. Stratified randomization for clinical trials. J Clin Epidemiol. 1999;52(1). 10.1016/S0895-4356(98)00138-3. 10.1016/s0895-4356(98)00138-3 9973070

[19] Kang H. Sample size determination and power analysis using the G*Power software. J Educational Evaluation Health Professions. 2021;18. 10.3352/JEEHP.2021.18.17. 10.3352/jeehp.2021.18.17 PMC8441096 34325496

[20] Fujioka-Kobayashi M et al. ‘Improved growth factor delivery and cellular activity using concentrated platelet-rich fibrin (C-PRF) when compared with traditional injectable (i-PRF) protocols’, *Clinical Oral Investigations*, vol. 24, no. 12, 2020, 10.1007/s00784-020-03303-7. 10.1007/s00784-020-03303-7 32382929

[21] Blanco J, Alonso A, Sanz M. Long-term results and survival rate of implants treated with guided bone regeneration: a 5-year case series prospective study. Clin Oral Implants Res. 2005;16(3). 10.1111/j.1600-0501.2005.01106.x. 10.1111/j.1600-0501.2005.01106.x 15877749

[22] Rogers GF, Greene AK. Autogenous bone graft: Basic science and clinical implications. J Craniofac Surg. 2012;23(1). 10.1097/SCS.0b013e318241dcba. 10.1097/SCS.0b013e318241dcba 22337435

[23] Yewale M, Bhat S, Kamath A, Tamrakar A, Patil V, Algal AS. Advanced platelet-rich fibrin plus and osseous bone graft for socket preservation and ridge augmentation – A randomized control clinical trial. J Oral Biology Craniofac Res. 2021;11(2). 10.1016/j.jobcr.2021.01.016. 10.1016/j.jobcr.2021.01.016 PMC7900600 33665072

[24] Clark D, et al. Advanced platelet-rich fibrin and freeze-dried bone allograft for ridge preservation: a randomized controlled clinical trial. J Periodontol. 2018;89(4). 10.1002/JPER.17-0466. 10.1002/JPER.17-0466 PMC6483085 29683498

[25] Stella E, Wahyuningsih KA. Perbandingan Perubahan Luas Luka dan Angiogenesis pada Luka Bakar Derajat IIB Tikus Sprague Dawley yang Diberikan Advanced platelet-rich Fibrin dan Advanced platelet-rich Fibrin Plus. Jurnal Kesehatan Andalas. 2021;10(2). 10.25077/jka.v10i2.1616.

[26] Giudice A, Esposito M, Bennardo F, Brancaccio Y, Buti J, Fortunato L. ‘Dental extractions for patients on oral antiplatelet: a within-person randomised controlled trial comparing haemostatic plugs, advanced-plateletrich fibrin (A-PRF+) plugs, leukocyte- and plateletrich fibrin (L-PRF) plugs and suturing alone’, Eur J Oral Implantol, vol. 12, no. 1, 2019. 31116189

[27] Santos Pereira VB, Barbirato DDS, Do Lago CAP, Vasconcelos BCDE. The Effect of Advanced platelet-rich fibrin in tissue regeneration in reconstructive and graft surgery: systematic review’. J Craniofac Surg. 2023;34(4). 10.1097/SCS.0000000000009328. 10.1097/SCS.0000000000009328 37143188

[28] Kosmidis K, Ehsan K, Pitzurra L, Loos B, Jansen I (2023). An in vitro study into three different PRF preparations for osteogenesis potential. J Periodontal Res.

[29] Pitzurra L, Jansen IDC, de Vries TJ, Hoogenkamp MA, Loos BG. Effects of L-PRF and A-PRF + on periodontal fibroblasts in in vitro wound healing experiments. J Periodontal Res. 2020;55(2). 10.1111/jre.12714. 10.1111/jre.12714 PMC7154757 31782171

[30] Tiwari V, Agarwal S, Goswami V, Gupta B, Khiraiya N, Soni VR. Effect on injectable platelet rich fibrin in augmentation of thin gingival biotype. Int J Health Sci. 2022;6(S1). 10.53730/ijhs.v6ns1.4802.

[31] Gadallah S, Rafat A, Fadel M, Elgohary I, Sharshar A, Misk T. Clinical and histopathological studies on the efficacy of multiple injections of I-PRF Versus the single use of A-PRF in repair of Achilles Tendon rupture in dogs: an experimental study. J Curr Veterinary Res. 2022;4(2). 10.21608/jcvr.2022.267520.

[32] Avila-Ortiz G, Gubler M, Romero-Bustillos M, Nicholas CL, Zimmerman MB, Barwacz CA. Efficacy of Alveolar Ridge Preservation: a Randomized Controlled Trial. J Dent Res. 2020;99(4). 10.1177/0022034520905660. 10.1177/0022034520905660 32050833

